# Neural-level associations of non-verbal pragmatic comprehension in young Finnish autistic adults

**DOI:** 10.1080/22423982.2021.1909333

**Published:** 2021-05-23

**Authors:** Aija Kotila, Jussi Tohka, Jukka-Pekka Kauppi, Ilaria Gabbatore, Leena Mäkinen, Tuula M. Hurtig, Hanna E. Ebeling, Vesa Korhonen, Vesa J. Kiviniemi, Soile Loukusa

**Affiliations:** a Faculty of Humanities, Research Unit of Logopedics, University of Oulu, Oulu, Finland; b A. I. Virtanen Institute for Molecular Sciences, University of Eastern Finland, Kuopio, Finland; c Faculty of Information Technology, University of Jyväskylä, Jyväskylä, Finland; d Department of Psychology, University of Turin, Turin, Italy; e Clinic of Child Psychiatry, Oulu University Hospital and PEDEGO Research Unit, University of Oulu, Oulu, Finland; f Research Unit of Clinical Neuroscience, Psychiatry, University of Oulu; g Department of Diagnostic Radiology, Medical Research Center, Oulu University Hospital and Research Unit of Medical Imaging, Physics and Technology, the Faculty of Medicine, University of Oulu, Oulu, Finland; h Oulu Functional NeuroImaging-lab, Medical Research Center, Oulu University Hospital, Oulu, Finland

**Keywords:** Autism spectrum, autistic adults, pragmatics, non-verbal, video, fMRI

## Abstract

This video-based study examines the pragmatic non-verbal comprehension skills and corresponding neural-level findings in young Finnish autistic adults, and controls. Items from the Assessment Battery of Communication (ABaCo) were chosen to evaluate the comprehension of non-verbal communication. Inter-subject correlation (ISC) analysis of the functional magnetic resonance imaging data was used to reveal the synchrony of brain activation across participants during the viewing of pragmatically complex scenes of ABaCo videos. The results showed a significant difference between the ISC maps of the autistic and control groups in tasks involving the comprehension of non-verbal communication, thereby revealing several brain regions where correlation of brain activity was greater within the control group. The results suggest a possible weaker modulation of brain states in response to the pragmatic non-verbal communicative situations in autistic participants. Although there was no difference between the groups in behavioural responses to ABaCo items, there was more variability in the accuracy of the responses in the autistic group. Furthermore, mean answering and reaction times correlated with the severity of autistic traits. The results indicate that even if young autistic adults may have learned to use compensatory resources in their communicative-pragmatic comprehension, pragmatic processing in naturalistic situations still requires additional effort.

## Introduction

As part of neurodiversity, autism spectrum disorder (ASD) is a complex neurodevelopmental condition that is associated with atypical social interaction and communication in relation to typically developing individuals [1]. Inherited genetic influences significantly contribute to the incidence of ASD globally [2]. While the characteristics of autistic traits may change from childhood to adulthood [3], social challenges usually persist across the lifespan. Despite their intellectual abilities, young autistic adults often experience social isolation among their neurotypical peers [4]. Challenges in the area of social cognition, including understanding verbal or non-verbal social clues, can often lead to misunderstandings in communication situations and, therefore, can cause problems in relationships [5,6]. Pragmatic communication abilities, which develop in interaction with other people, refer to how language and non-verbal means, such as gestures, are understood and expressed in specific communication contexts [7]. A lack of pragmatic communication abilities clearly has a negative impact on one’s life [8], and can increase the likelihood for marginalisation. However, research concerning the pragmatic skills among young autistic adults is still scarce. Evidence exists suggesting that autistic persons have a different manner of processing information in order to make pragmatic inferences compared to their neurotypical peers [9,10], but more knowledge is needed about how the processing of pragmatically challenging scenarios is executed on the neural level.

Communicative intentions in social interactions are conveyed through linguistic and non-linguistic means [11]. Extralinguistic communication refers to the use of non-verbal communication such as gestures and facial expressions, which commonly accompany communicative acts. The interaction of multiple mental processes, including attention, memory, motivation, and emotion, but also the capabilities to perceive and integrate multimodal social cues, are required to appropriately process the pragmatic information related to communication [12]. There is evidence that autistic features affect how the processing of multimodal social cues is carried out, such as understanding emotions from facial expressions, or the reading of co-speech gestures and body language [13–18]. In addition, studies have found slower and less efficient processing of social information by autistic persons compared to neurotypical ones [19,20], which can in part explain longer response times in mental-state inference tasks [21]. Atypical pragmatic inferencing is often witnessed in autistic persons, especially when trying to comprehend social interaction requiring processing of communicative acts with high inferential load [22,23]. This may be related to, but not comprehensively explained by, theory of mind (ToM) skills [24,25].

The social brain includes several brain regions [26–28]. For example, the insula, which is believed to be part of the salience network (SN), has been linked to the detection of socially salient events and cognitive processing of empathy [29–31]. Earlier studies have demonstrated differences between autistic and neurotypical individuals in the processing of social cues, which are also visible on the neural level [32–34]. For instance, findings report atypical brain response to stimuli concerning face perception with emotional content [35–38]. Furthermore, studies have found atypical brain organisation and activity in autistic persons in brain regions related to social cognition and emotion processing [39–43]. Although studies have reported intact perception of simple social gestures or encoding basic biological motion in autistic persons [44,45], they have shown atypical brain activation when processing co-speech gestures [46]. However, little is known about pragmatic communication on the neural level, but some evidence exists indicating that pragmatic processing of language activates distinct neuroanatomical correlates [47]. A recent study suggests that there may be common areas processing pragmatic phenomena, but also areas that distinguish between different types of communicative acts [48].

The goal of the present study was to investigate the pragmatic comprehension skills and corresponding neural-level findings in young adults from Northern Finland who had been diagnosed with ASD as a child and neurotypical (NT) controls. The present study is part of a longitudinal autism spectrum research carried out at the University of Oulu and the Northern Ostrobothnia Hospital District which is the northernmost of the five university hospital districts in Finland. As a novel method in pragmatic research, we used naturalistic video presentations that convey more multimodal cues related to pragmatic understanding than still pictures [see also 49]. Since we wanted to assess both behavioural and neural-level functioning, a behavioural investigation was done using a selection of video-based items of the Assessment Battery for Communication [ABaCo; 50]. The first aim of this study was to find out if there was a difference between the autistic and control groups in non-verbal comprehension when measured using extralinguistic comprehension items of the ABaCo test. To gain more knowledge about how socially and pragmatically challenging situations were processed, the reaction and answering times to ABaCo questions were also measured. Therefore, the second aim was to explore whether there was a difference between the autistic and control groups in ABaCo reaction and answering times, or whether these times correlated with autistic traits. Since we were also interested in the neural processing of pragmatic communicative situations, the third aim of the study was to assess the differences in the synchrony of the neuronal network activity between groups while the subjects viewed the videos from ABaCo. The brain data were acquired using functional magnetic resonance imaging (fMRI), which can provide temporal signals of brain oxygenation with blood oxygen level-dependent (BOLD) contrast, indirectly revealing the fluctuations and alterations of brain activity. The neural correlates of test stimulus viewing were analysed using the inter-subject correlation (ISC) analysis method [51,52]. Compared with other methods, the ISC analysis of fMRI data is an effective and unique tool for identifying brain areas that activate in a synchronous manner within a group of viewers during a continuous stream of visual stimulation [53,54]. Based on ISC, differences in synchronised processing between groups can be revealed. By comparing the behavioural and neural-level findings, this study increases understanding about how young autistic adults process pragmatic communicative situations, which is important for arriving at most beneficial support strategies.

## Methods

### Participants

The participants of this study were nineteen young adults from Northern Finland who had been diagnosed with ASD when they were children (14 male, mean age 23.6, range 19–31, *SD* 3.3), and nineteen control participants (15 male, mean age 22.7, range 19–29, *SD* 2.2). This study belongs to a multidisciplinary project at the Oulu University Hospital and the University of Oulu called “Autism spectrum disorders – a follow-up study from childhood to young adulthood”. The project originally begun when the participants were young children, and later they were re-recruited during 2014–2015 for clinical and fMRI assessment from which data was collected for the present study. Participants were drawn from two longitudinal studies: a community-based study in the Northern Ostrobothnia Hospital District in Finland which began in 2000 [55] and a clinic-based study at Oulu University Hospital, Finland (OYS) which began in 2003 [56,57]. Diagnoses of ASD participants were confirmed based on the International Classification of Diseases–10th Revision [75] criteria by a trained diagnostician clinical psychologist and a medical doctor (a paediatrician or a child psychiatrist) after careful clinical assessment [for more detailed description of the diagnostic procedure, see 55, 56, 58]. The diagnostic confirmation was supported by the usage of the Autism Diagnostic Interview-Revised (ADI-R) [59] and Autism Diagnostic Observation Schedule (ADOS) [60]. The age range, when the diagnosis was confirmed, was 7–19 years. Control participants were originally recruited in 2006 [56]. Participants in the present study had no intellectual disability and there was no significant difference between groups in general ability index (GAI) of WISC-III (Autistic group: *M* = 111.3(13.2), range = 84–131; Control group: *M* = 103.7(13.0), range = 83–126) [61]. During the re-recruiting in 2014–2015, the autistic traits of the participants were assessed using the Finnish version of Autism Quotient (AQ) questionnaire [62]. There was a significant difference (*U* = 217.5, *p* = .002) between the autistic (*M* = 19.7(9.0), *Md* = 19.0, missing 2) and control (*M* = 10.6(4.9), *Md* = 10.0, missing 3) groups in AQ scores. Based on the study in [63], the mean AQ value in Finnish autistic individuals (*n* = 52) was 22.5(8.3) and in controls (*n* = 1686) 13.1(6.4). The cut-off scores in the Finnish sample (men 18, females 16) were lower than in an English sample [e.g. 62], probably due the cultural differences as discussed in [63]. Research was approved by the Ethical Committee of Medical Research in the Northern Ostrobothnia District of Finland. A written informed consent was obtained from the participants prior to behavioural and fMRI phases of the study.

### Behavioural assessment

To assess non-verbal extralinguistic comprehension, we used a selection of items from form A of the Assessment Battery for Communication [ABaCo; 50, 64], a validated tool for pragmatic communicative abilities evaluation, that was recently translated and adapted to Finnish [113] . In particular language-free items of the Finnish version were used for the assessment of pragmatic comprehension skills (Table 1). In these six selected items, the focus is on the comprehension of simple communicative acts expressed through the use of gestures only. Each item consists of a video presenting a communicative act. Immediately after watching each video, the subjects were asked questions defined in the ABaCo test battery concerning the pragmatic content of the clip. The recorded answers were later transcribed and answers were rated according to the test scoring procedure: correct answers scored ‘1ʹ and incorrect ‘0ʹ and ratio of correct answers was calculated for each test category. Reaction and answering times to questions were measured.

**Table 1.** Stimulus data of the ABaCo items

Item	Communicative act	Description	Duration [s]
X9	request	The actor is carrying a tray of chocolates. He holds it out for the spectator, with a questioning in the air, as if to ask “*Would you like some too*?”	27
X1	statement	There is a cake in front of the actress who gives a thumb up sign, as if to say “*It’s good!*”	11
X10	request	The actor rubs his hands up and down his arms, as if to say “It’s so cold!”. He looks at the open window beside him making a closing movement with his hands, as if to ask “*Could you close it, please?*”	22
X8	question	A tourist, who wants to visit a certain part of the town, is holding a map because he doesn’t know how to get there. He turns to the spectator pointing a specific point as if to ask: “*How do I get here?*”	22
X16	order	A man is pointing at a chair by a set table indicating very firmly as if to say “*Sit down!*”	25
X4	statement	The actor is sitting on a chair and with one hand on his forehead looking towards the camera with an air of suffering, as if to say “*I’m so tired!*”	25

### Acquisition of fMRI data

The fMRI data were acquired on a Siemens Skyra 3 T scanner using a 32-channel head coil and echo-planar imaging (EPI) pulse sequence with the following parameters: TR = 2150 ms, TE = 28 ms, flip angle = 15°, 3 mm cubic voxel, matrix size 64 × 64. A total of 45 axial slices were sampled for whole-brain coverage. For each participant, in total 430 volumes of BOLD data were taken, of which brain volumes 92–153 comprise the data in this study. Furthermore, Anatomical 3D T1_MPRAGE (TR = 1900 ms, TE = 2.49 ms, TI = 900 ms, flip angle = 9°, FOV = 240 mm, 0.9 mm cubic voxel) was taken from every participant.

An introductory video and also written material was prepared describing the scanning procedure. All autistic and some control participants saw the introductory video before entering the scanner. The autistic participants were familiar with the scanning procedure, since their brains had been scanned 10 years previously as part of another study. To minimise scanning time, the stimulus video clips were merged following each other in a consecutive manner. The stimulus video was shown to participants on an MRI-compatible screen while they were laying in the scanner. The participants wore ear protection while the voice of the video could be heard through a hole in the earplug, volume being adjusted to comfortable hearing level.

The stimulus in fMRI scanning consisted of the same ABaCo videos as the behavioural testing to allow for comparison between behavioural and neural-level performance. All the participants completed the behavioural part of the ABaCo a minimum of 24 hours before entering the scanner. Videos were presented in the same order as during the structured ABaCo testing and in one continuous run. The order and content of the videos is presented in Table 1. The total duration of the stimulus data was 2 min 12 s corresponding 62 brain volumes.

### Preprocessing of fMRI data

Participants’ BOLD image series were preprocessed using a typical FMRIB software library (FSL) pipeline [65]. The preprocessing stages included removal of the skull (BET), motion correction (MCFLIRT), brain registration to a common MNI-space, spatial smoothing and high-pass filtering. The data were spatially smoothed using Gaussian filtering based on 5 mm full width at half maximum. The timeseries in each voxel was high-pass filtered with a cut-off frequency of 0.008 Hz. No significant differences were found between the groups in relative or absolute head motion values.

### Statistical analysis

Analysis of the fMRI data was done in the Matlab (R2017b, MathWorks, Natick, MA, USA) environment using the ISC toolbox [52]. In the ISC analysis originally proposed in [51], correlation coefficients between fMRI time series of the subjects are calculated in the corresponding brain locations, and statistical inference is performed to construct brain maps describing extent of shared processing across subjects in different brain areas. A major benefit of using the ISC-based approach is that experimental stimuli need not to be explicitly modelled and therefore fMRI data acquired under naturalistic stimuli, like movies, can be easily analysed.

Only grey matter voxels of the brain were included in the ISC computations. For the extraction of the grey matter voxels, a mask was created where white-matter, ventricles and brain-stem were excluded from the full brain volume based on a Harvard-Oxford cortical atlas.

The basic ISC analysis [52,66] provides information about the extent of shared processing across the participants *within* a group of subjects. Here, it was used for obtaining group-level ISC maps for the autistic and control groups separately. Given a group of *N* subjects, a group-level ISC statistic for voxel *j* was computed as:

$${\overset{ˉ}{r}}_{j}=\frac{1}{N\left(N-1\right)/2}\sum_{n=1}^{N}\sum_{m=2,m>n}^{N-1}r_{j}\left(n,m\right)$$

where ${\overset{ˉ}{r}}_{j}$ denotes an average ISC of voxel *j* across all *N*(*N*-1)/2 subject pairs, and *r_j_(n,m)* is a Pearson’s correlation coefficient of the fMRI time-series between subjects *n* and *m* for voxel *j*. After computing average ISC statistics for each voxel, a non-parametric voxel-wise bootstrap resampling test was run with 1 000 000 realisations [52,66]. Obtained *p*-values were corrected for multiple comparisons by the false discovery rate (FDR) using the Benjamini-Hochberg procedure [67]. The basic ISC analysis has been shown to produce activation maps closely matching those of the standard GLM-based analysis when the stimuli are simple and can be modelled [68].

Next, ISCs between the groups were compared by computing ISC difference maps showing differences in ISCs between the autistic and control groups [69]. A studentized SAM statistic was considered in this study [70,71]. A permutation test was performed based on an ISC matrix of size (*N_ASD_* + *N_NT_*) x (*N_ASD_* + *N_NT_*) for each voxel, consisting of both within- and between-group ISCs [72]. Rows and columns of these matrices were randomly permuted 15 000 times (subject-wise permutations). The voxels with low p-values (*p* < .05) were entered for additional permutation iterations for increased accuracy of the *p*-values. Storey’s procedure was used for multiple comparison correction to control for the FDR [73,74]. The ISC brain maps were viewed in the visual GUI of the ISC 3.0 toolbox using the MNI-152 stereotactic template (https://www.nitrc.org/projects/isc-toolbox/).

SPSS was used for statistical analysis of behavioural results. Because assumptions of normal distribution were not met, non-parametric Mann-Whitney *U* test was applied to analyse group differences for ABaCo scores. Repeated measures ANOVA was used to explore group differences for reaction and answering times. In further analysis, the Moses extreme reaction test with the outliers trimmed option was used to test if extreme values of mean reaction or answering times were equally likely in both groups. In addition, Pearson correlation test was used to assess relationship between a person’s mean reaction or answering time and AQ score.

## Results

### Non-verbal extralinguistic comprehension scores

According to the Mann-Whitney *U* test there was a trend towards a significant difference (*U* = 120.5, *p* = .060, *r* = .31) between groups for ABaCo scores where median result for the autistic group (*n*= 19, *Mdn *= .83, range = .33–1.00) was lower than for the control group (*n* = 19, *Mdn  *= 1.00, range  = .67–1.00). Ceiling effect impacted the results in the control group but not in the autistic group. The percentage of correct answers in groups for each item (Table 2) shows that comprehension of item X10 (request) differentiated the groups most. Moses extreme reaction test found that scores were more dispersed (*p* = .003, outliers trimmed) in the autistic than control group. There were two participants in the autistic group who were performing worse than the weakest performers of the control group (score < .67).

**Table 2.** Correct answers for ABaCo items within each group

		**Correct answers (%)**	
**Item**	**Communicative act**	**Autistic** **group**	**Control** **group**
X9	request	94.7	100.0
X1	statement	78.9	89.5
X10	request	52.6	89.5
X8	question	78.9	84.2
X16	order	84.2	89.5

### Answering and reaction times in behavioural testing

**Figure 1.**
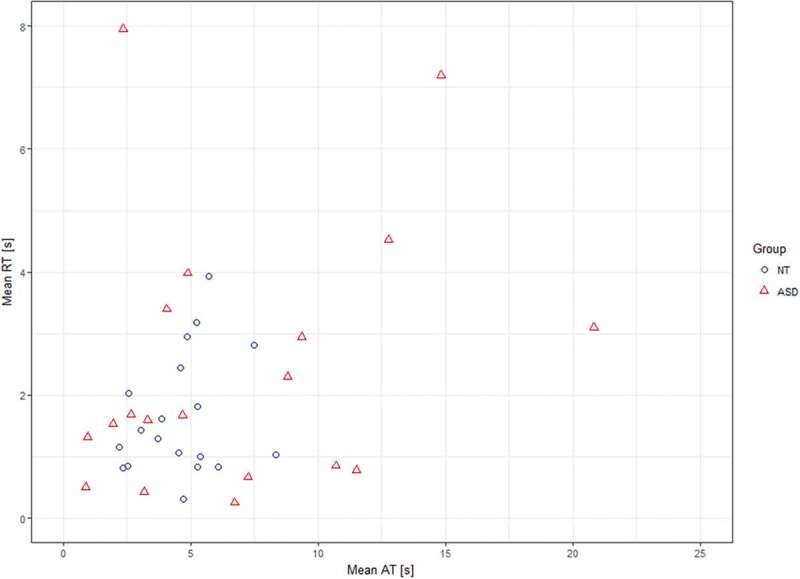
Mean answering time (AT) versus mean reaction time (RT) per participant in the autistic (ASD) and the control (NT) groups

When comparing answering times to behavioural ABaCo items repeated measures ANOVA (*F*(1, 36)  = 3.24, *p* = .080, *ƞ^2^ *= .082, *r* = 0.29) did not reveal statistical difference between the autistic (*M* = 6.9 s, range = 0.4–40.9, *SD *= 7.9) and the control (*M* = 4.6 s, range = 1.1–19.8, *SD *= 4.6) group. However according to Levene’s test, groups had unequal variances (*p*< .05) for answering times and in further analysis the Moses extreme reaction test revealed that participants’ mean answering times were more dispersed (*p* < .001) in the autistic than control group (Figure 1). There was a subgroup of 6 autistic individuals whose mean answering times exceeded *M*+ 1*SD* (9.2 s) value of the control group. In addition, relationship between a participant’s mean answering time and AQ score was assessed. A Pearson correlation test showed that the two were correlated (*r* = .410, *p* = .018, *N* = 33) revealing that longer answering time was related to more severe autistic traits.

Repeated measures ANOVA (*F*(1, 36) = 2.13, *p* = .153, *ƞ^2^ *= .056, *r* = 0.24) did not reveal statistical difference in reaction times to behavioural ABaCo items between groups (Autistic group: *M* = 2.5 s, range = 0.0–27.4, *SD *= 3.9; Control group: *M* = 1.7 s, range = 0.0–12.9, *SD *= 2.1). According to Levene’s test, groups had unequal variances (*p* < .05) for reaction times. Further analysis (Moses extreme reaction test) showed that there was no statistical difference (*p* = .116) in range of mean reaction times between the groups (Figure 1). However, mean reaction times and AQ scores were positively correlated (*r* = .380, *p* = .029, *N* = 33).

### fMRI results

**Figure 2.**
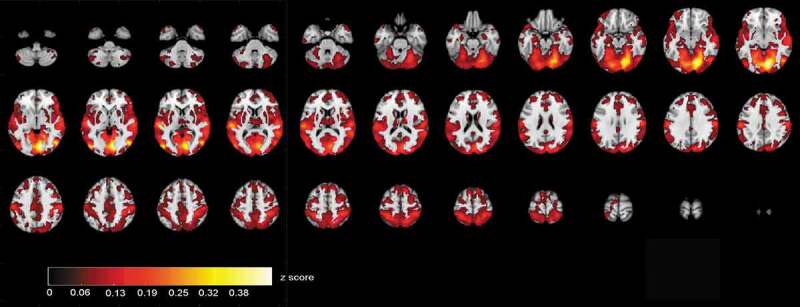
Correlating activation areas in the control group (*p*< .001, FDR corrected)

**Figure 3.**
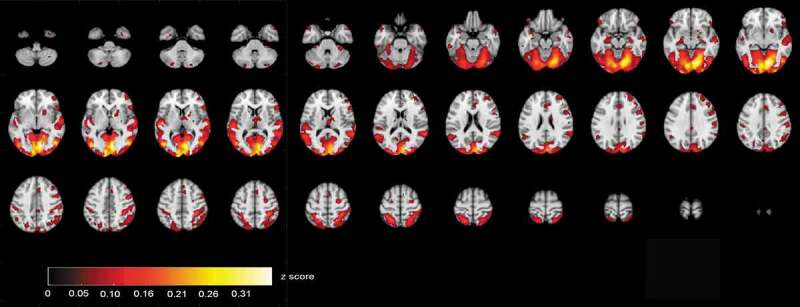
Correlating activation areas in the autistic group (*p*< .001, FDR corrected)

ISC maps of brain activity in groups were calculated from fMRI data that were acquired when the participants were viewing videos of the selected ABaCo items. ISC maps (*p* < .001, FDR corrected) showed more ISC in the control group most synchronous activity being right dominantly in the visual cortex and also in temporal and frontal areas (Figure 2). In comparison, ISC map in the autistic group shows that synchronous activity tends to be located mostly in the visual rather than frontotemporal areas in general (Figure 3).

**Figure 4.**
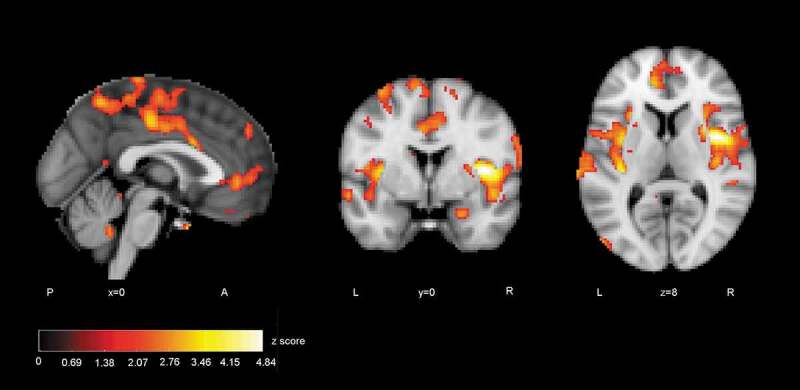
ISC group comparison (control > autistic, p < .05, FDR uncorrected)

**Figure 5.**
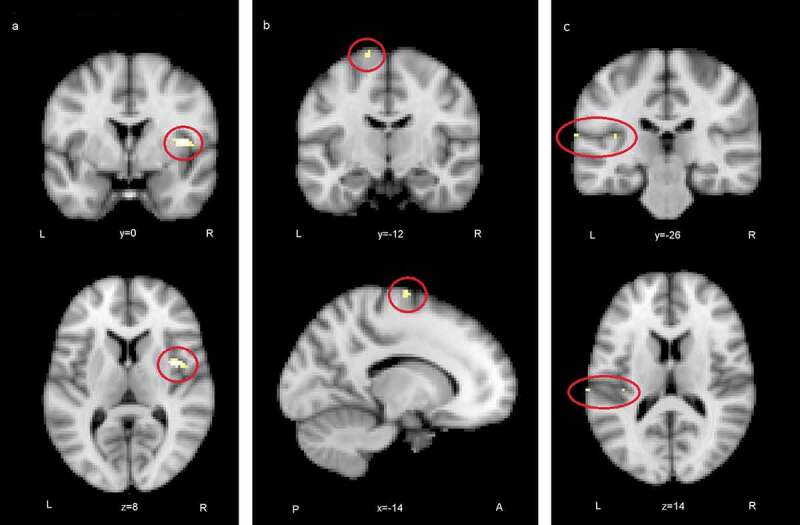
Regions where ISC analysis yielded highest statistics between groups (control > autistic): a) the right anterior insula, b) the left superior frontal gyrus, c) the left secondary somatosensory cortex and the posterior insula (FDR corrected)

ISC difference maps were calculated between the autistic and control groups to reveal statistically significant regions of different correlations between groups. The difference map showed several areas where correlation of activity was greater in the control than autistic group (Figure 4). At *p*< .05, uncorrected level ISC maps revealed distributed areas of higher correlation in the control group including more the bilateral insular and midline cingulate cortex typical to Salience network (SN). Also, ventromedial frontal areas of the Default mode network (DMN) had higher synchrony in the control than autistic group. The difference map after voxel-wise FDR correction (*p* < .05, FDR corrected threshold at *p*= .000026, z = 4.20) showed significantly greater ISC in the control group in regions depicted in Figure 5. The location information of clusters of peak significance of ISC difference (control group > autistic group) is presented in Table 3.

**Table 3.** Locations of clusters of peak significance in group comparison (control group > autistic group) in ABaCo extralinguistic items

**Anatomical regions**	**MNI (2 mm) coordinates** **Max. values**			**Cluster size**	**z value**
				**(voxels)**	**(max)**
**control group > autistic group**	**x**	**y**	**z**		
**R. central opercular cortex, anterior insula**	42	0	8	51	4.84
**L. superior frontal gyrus, premotor cortex**	−14	−10	74	7	4.62
**L. secondary somatosensory cortex, parietal operculum**	−68	−26	14	2	4.36
**L. secondary somatosensory cortex, parietal operculum, posterior insula**	−38	−26	14	2	4.25

_R=right, L=left_

## Discussion

### Behavioural results

A comparison between the autistic and control groups on the behavioural level yielded a medium effect size for the items measuring non-verbal extralinguistic comprehension, with a ceiling effect in the control group. Even though there was no statistically significant difference between the groups in ABaCo items, the test scores were significantly more dispersed in the autistic group suggesting that individuals who were diagnosed with ASD when they were children may still have some challenges in non-verbal pragmatic comprehension in their young adulthood [22]. The issue may be related to the ability of the individuals to understand a communicative act with respect to a particular communicative context. While the perception of communicative gestures may be intact in structured situations, the processing of pragmatic information in naturalistic settings requires additional mental effort for autistic persons, and comprehension of sophisticated pragmatic tasks can be challenging [19,76,77]. One can argue that the autistic participants may have experienced more cognitive load in the extralinguistic comprehension task, which possibly has impacted the identification of facial expressions and gestures [78]. According to some studies, autistic persons show decreased accuracy in processing social information from facial expressions, especially information related to eye gaze [33,79].

In addition, the results suggest that there is variability in respect to pragmatic skills in autistic persons [80]. Furthermore, despite a correct inferencing outcome, autistic persons may have used compensatory mental processing in understanding expressions shown in the videos [81, 82]. After viewing the videos, some individuals in the autistic group mentioned out-of-context details from the scene, which accords with earlier studies [21]. Even if detailed visual processing may be a strength in autism [83, 84] there is evidence that school-aged intellectually able autistic children are not as capable at using contextual information in pragmatic tasks as their typically developing peers [85]. The results also lend credence to the notion that some autistic individuals may have had greater development of their pragmatic skills, probably due to such factors as a supportive developmental environment. Different developmental trajectories are possible after childhood ASD diagnosis and some individuals can gain social skills comparable to non-autistic individuals by adulthood, possibly also due to the dynamic reconfiguration of brain networks during development [86]. Interestingly, shared environmental factors have been reported to contribute to occurrence of autism more in Finland than in other Nordic countries i.e. Sweden and Denmark [2].

The results indicated that the long mean answering time to ABaCo’s questions about the pragmatic content of the video clips is related to more prominent autistic traits. Although a comparison between the groups did not show a difference in answering times, a comparison for extreme values in mean answering times showed a significant difference between groups, with the values being more dispersed across individuals in the autistic group. There was a group of 6 individuals in the autistic group whose values exceeded *M + 1SD* values of the control group. The result suggests that performance in the autistic group is not uniform in tasks requiring pragmatic processing and individuals may have different levels of processing skills [6,87]. Individuals with long answering times may use more cognitive effort, together with compensatory strategies, in the comprehension of social situations and in providing their answers than others in the group [6,21,88,89]. The long answering times of some individuals in the autistic group may also reflect either dysfluent speech or verbose communication style. Especially the verbose style may help mediate the explicit processing of social situations [80]. Further work is required to investigate the contents of the answers in the autistic group concerning topic relevancy and coherency to gain a better understanding of the underlying mental processes.

On the group level, there was no statistically significant difference between the groups in reaction times. This is in line with some other studies that have not found any differences between autistic and control groups in reaction times when processing facial emotions [33,35]. However, in this study, there was a relationship between a person’s mean reaction time and autistic traits, in which a longer time suggested more prominent traits. Long reaction time may mean that deriving an answer from the context is not as automatic as it typically is, and a person has to use additional cognitive effort in order to understand what the other person is trying to convey [81,90].

### fMRI results

In group comparisons, the ISC difference map for extralinguistic items showed several areas where the correlation of brain activity was significantly greater within the control than autistic group, revealing that activity reached synchronisation in wider brain areas. Areas of greater ISC in the control group included the bilateral insular and midline cingulate cortex regions that are typical to the SN [29]. The stimulus also allowed more ISC in the ventromedial frontal regions in the control than autistic group. These regions have been linked to the DMN [91]. The atypical recruitment of SN and DMN has been associated with autistic traits [79, 92–94]. After stringent FDR correction, significant areas of difference between groups were the right anterior insula and opercular cortex, left superior frontal gyrus (SFG) extending to the premotor cortex and left secondary somatosensory (auditory) cortex together with the parietal operculum and posterior insula. The activation of these areas was more correlated in the control group, probably showing that brain activity becomes synchronised if viewers assume similar mental perspectives during stimulus viewing [54, 95, 96]. The finding suggests perhaps weaker modulation of brain states for varying stimuli in the autistic group [97]. The results are similar to the study reported in [82], where the participants of the autistic group of the present study were young teenagers.

When viewing the ABaCo items, the control group was more homogeneous in insula activation patterns than the autistic group. The result is in line with previous studies suggesting a relationship between atypical insula activation and autistic traits [33,38,89]. In the present study, the activity of the right insula was significantly less correlated within the autistic group. A link between autistic traits and the right insula has been demonstrated also in some earlier studies [82,98,99]. It has been found that stimulus related to mental imagery and emotive gesture stimuli increases activity in the right anterior insula [100,101]. An atypical right anterior insula response in the autistic group may be related to deviant mental resonance for shown situations requiring implicit decoding of facial expressions and gestures [35,38,102–104]. The anterior insula is an area that has also been associated with integrating salient external stimuli to mental processes and interpreting goal-directed actions [105,106]. In another study, the fronto-insular network reacted to emotions when subjects viewed movie clips [107]. The insula has also been linked to the pragmatic processing of language [47].

On the left side of the brain, the ISC difference map included the SFG and premotor cortex, secondary somatosensory (auditory) cortex, parietal operculum, and posterior insula. According to previous studies, attributing mental states to other people activates the SFG and premotor regions [108,109] and the activation of these areas is reduced in autistic persons [110]. The secondary auditory cortex, parietal operculum, and posterior insula respond to the affective content of sound independent of the modality [111]. The videos did not include speech, but they did include sounds of actions that could be interpreted representing different vitality forms, the perception of which has been found to be different between typically developing and autistic children [105,112]. Perhaps greater ISC in these regions in the control group can be explained by attention to the multimodal emotive signals.

The data present only a narrow sample of an autistic group because of the longitudinal and also voluntary nature of the study. Larger group sizes would have yielded more reliable results. Also, the autistic participants had rather good general abilities. Further studies with larger sample sizes as well as different ability and gender profiles should be warranted. In this study, the length of the fMRI sample was rather short. However, in case of the ISC analysis, a longer fMRI sample is not necessarily better, since even a relatively short sample can allow for reliable ISC results, if it includes enough neural-level reactions [52]. In addition, a relatively short acquisition time ensured participants’ vigilance during the scan. Furthermore, the autistic participants were not diagnosed again in this follow-up study as adults, based on the general assumption that ASD is a permanent condition. However, as part of the follow-up assessment, the AQ scores were collected and compared between the groups, showing significantly more autistic traits in the autistic group.

### Conclusions

In this study, naturalistic video presentations were used to assess non-verbal pragmatic comprehension abilities in young adults who have been diagnosed with ASD in their childhood in the Northern Ostrobothnia Hospital District in Finland. The ISC analysis method was proven to be effective in revealing differences in synchronised neuronal network activity between the autistic and control groups. Even though the performance between young autistic adults and control participants was similar at the behavioural level in non-verbal pragmatic comprehension tasks, the findings indicate differences in the synchrony of brain activity between the groups when watching the related video clips. The results confirmed the earlier results of atypical neural-level functioning in autism, but also showed that the atypical functioning is related to the comprehension of extralinguistic communicative signals, including non-verbal gesturing and facial expressions in naturalistic social situations. We suggest that the deviant processing of pragmatic cues in autism may be linked to abilities to modulate brain activity in accordance with specific communicative situations. Some young autistic adults, but not all, have learned to utilise compensatory strategies to overcome these pragmatic challenges, but the processing of social situations may require additional effort.

### Implications

The results indicate that there is variability concerning pragmatic skills in autistic persons, and for some individuals, challenges continue until young adulthood. These individuals should be identified for tailored support services to prevent possible social difficulties and even marginalisation. Since social challenges are often bidirectional, peer education about differences in pragmatic processing style should be included in support services. Even though contextual processing in this study took more time for autistic persons than for the controls, the answers showed that autistic persons were able to use contextual information in most instances. This means that we need to educate the communication partners of autistic persons that they should try to reduce the tempo of discourse situations and allow more time for processing in order to make the communication environment pleasant for autistic persons. Furthermore, the communication partners should ensure that the extralinguistic signals that they are using are clear enough. In addition, the strengths of autistic persons should be taken into account when designing strategies to address pragmatic communicative challenges.
